# ASSOCIATIONS OF MEAN AND VARIABILITY OF ACTIGRAPHIC SLEEP AND GENERAL COGNITIVE PERFORMANCE

**DOI:** 10.1093/geroni/igae098.0248

**Published:** 2024-12-31

**Authors:** Linying Ji, Yuqi Shen, Orfeu Buxton, May Beydoun, Alan Zonderman, Michele Evans, Alyssa Gamaldo

**Affiliations:** Montana State University, Bozeman, Montana, United States; The Pennsylvania State University, University Park, Pennsylvania, United States; The Pennsylvania State University, University Park, Pennsylvania, United States; National Institute on Aging, Baltimore, Maryland, United States; National Institute on Aging, Baltimore, Maryland, United States; National Institute on Aging, Baltimore, Maryland, United States; Clemson University, Clemson, South Carolina, United States

## Abstract

Sleep health is associated with cognitive function in midlife and older adults. Little research investigated the relationship between cognitive performance and variability, in addition to mean sleep measures. Using a subsample HANDLSleep study (n=81; 72% female, 52% Black adults; age range: 46-81), this study examined the cross-sectional relationship between cognitive function and both variability and mean sleep with a Bayesian variability model. Cognitive function was measured using Montreal Cognitive Assessment (MoCA). Sleep measures were estimated using one week of wrist actigraphy (mean ± SD = 6.8 ± 0.4 day), including nighttime sleep time (TST), wake after sleep onset (WASO), nighttime sleep midpoint (Timing), Sleep maintenance efficiency (SMEff), and nap minutes (NAP). All models controlled for age, race, sex, WRAT3 literacy scores, and poverty status. Results showed that more variability in sleep Timing (b = -2.75, CI [-5.18, -0.45], p = 0.02) and longer mean nap minutes (b = -3.58, CI [-6.52, -0.93], p = 0.006) were associated with worse cognitive performance (lower MoCA score). No significant associations were observed for TST, WASO, SMEff and MoCA. Findings suggest the importance of considering both variability and mean sleep measures in understanding how sleep relates to cognitive function in middle-age and older adults.

